# Grade II whiplash injuries to the neck: what is the benefit for patients treated by different physical therapy modalities?

**DOI:** 10.1186/1754-9493-3-2

**Published:** 2009-01-16

**Authors:** Christoph Dehner, Martin Elbel, Philipp Strobel, Matthias Scheich, Florian Schneider, Gert Krischak, Michael Kramer

**Affiliations:** 1 Center of Surgery, Department of Orthopedic Trauma, Hand-, Plastic- and Reconstructive Surgery, University of Ulm, Steinhövelstrasse 9, 89075 Ulm, Germany; 2 Ulmkolleg School for Physiotherapists and Masseurs, Ulm, Oberberghof 5, 89081 Ulm, Germany

## Abstract

**Background:**

In a majority of cases, whiplash injuries are a domain of conservative therapy. Nevertheless it remains unclear whether physical therapy is of medical or economic benefit in patients with whiplash injuries.

**Methods:**

Seventy patients with acute Quebec Task Force (QTF) grade II whiplash injuries were randomized to two therapy groups and received either active (APT) or passive (PPT) physical therapy. Patients were compared with regard to pain and range of motion with data obtained in an earlier study from a group with grade II whiplash injuries in which the therapy recommendation had been "act as usual" (AAU; n = 20). The above-mentioned parameters were assessed at 24 hours and two months after the injury. Furthermore patients' period of disability was documented after two months.

**Results:**

After two months, patients in both the APT and PPT groups showed significant improvement in the median period of disability (active: 14 days; passive: 14 days) compared to the AAU group (49 days). No group difference was observed with regard to median improvement in range of motion (active: 120°; passive: 108°; activity as usual: 70°). The median pain reduction was significantly greater in the APT group (50.5) than in the PPT (39.2) or AAU group (28.8).

**Conclusion:**

Our data show that active physical therapy results in enhanced pain reduction and shortening of post-injury disability. Therefore, active physical therapy should be considered the treatment of choice in patients with QTF grade II whiplash injuries.

**Trial registration:**

The study complied with applicable German law and with the principles of the Helsinki Declaration and was approved by the institutional ethics commission.

## Background

The term "whiplash" in connection with motor vehicle collisions was first used by Gay and Abbott in 1953 to describe the whip-like hyperextension with subsequent hyperflexion as a result of a rear-end collision [1]. Meanwhile several studies simulating whiplash, describe three reproducible phases of head-neck kinematics [2-7]. In the first phase, the cervical spine shows a S-shaped curvature in which the more cranial motion segments undergo flexion, coupled with extension in the more caudal segments. It is supposed that injuries mainly located in the lower cervical spine are caused in this vulnerable phase. In the second phase, all segments of the cervical spine become extended, followed by a third phase in which the cervical spine passes once again through the initial position to finally reach maximum flexion.

Whiplash injuries represent one of the most common types of trauma in this age of increasing individual traffic mobility and their incidence continues to rise [8-10]. After a complaint-free interval of a few hours to one day (five hours, on average), 47 – 88% of patients report pain in the neck. [11-13] To describe the most determinant clinical symptoms, the Quebec Task Force (QTF) developed 1995 a classification system which allows a good assessment of the severity of the injury (table 1) [14]. In cases of QTF I° and II° whiplash injuries, the posttraumatic treatment is a domain of conservative therapy. Therapeutic measures have been exhaustively studied and compared [14-16]. Physical therapy has been assessed predominantly with respect to its effects on pain intensity and improving patients' range of motion. It seems that its efficacy is limited to a certain degree of improvement of these parameters in the acute stage of convalescence [17].

**Table 1:** Clinical classification of whiplash-associated disorders according to the Quebec Task Force

**QTF Grade**	**Clinical Symptoms**
**0**	**No complaint **about the neck, no physical signs
**I**	Neck complaint of pain, stiffness or tenderness only, **no physical signs**
**II**	Neck complaint and musculoskeletal signs *
**III**	Neck complaint and neurological signs **
**IV**	Neck complaint and **fracture or dislocation**

* Musculoskeletal signs include decreased range of motion and point tenderness; ** Neurologic signs include decreased or absent deep tendon reflexes, weakness and sensory deficits; Symptoms and disorders that can be manifest in all grades include deafness, dizziness, tinnitus, headache, memory loss, dysphagia and temporomandibular joint pain

The quality of past studies, however, has been criticized and the therapeutic recommendation to "act as usual" has been considered adequate for comparable therapeutic success [14,16]. There is, therefore, the overall impression that, compared with a spontaneous clinic course, physical therapy results in no statistically measurable advantage and the costs associated with physical therapy are not justified. It is important to note, however, that the effect of physical therapy in whiplash associated disorders has only been investigated in mixed QTF I and II populations [14-16]. Considering the better prognosis of QTF I compared to QTF II injuries [18], it is probable that the therapeutic outcome of previously conducted therapy studies constitute a false-positive evaluation of the QTF II sub-populations.

Because 84.5% of the costs due to whiplash injuries are caused by the 38.5% of patients whose absence from work lasted more than two months [14], the aim of acute therapy in view of cost saving must be to achieve the maximum reduction in healing time and thus reduce the period of disability. To date, only three therapy studies have addressed the "period of disability" as an outcome parameter [19-21]. The results of these studies, however, are contradictory and it is unsolved to what extent therapeutic measures may reduce the period of disability and contributes to cost savings.

In summary it remains unclear whether physical therapy is of medical or economic benefit in patients with whiplash injuries. The present study is, to our knowledge, the first to simultaneously compare the efficacy of two physical therapy regimens and the recommendation to "act as usual" on the basis of clinical (pain intensity, range of motion) and economic (period of disability, sickness costs) outcome parameters in patients suffering acute whiplash injury.

## Materials and methods

### Study design

**Figure 1:**
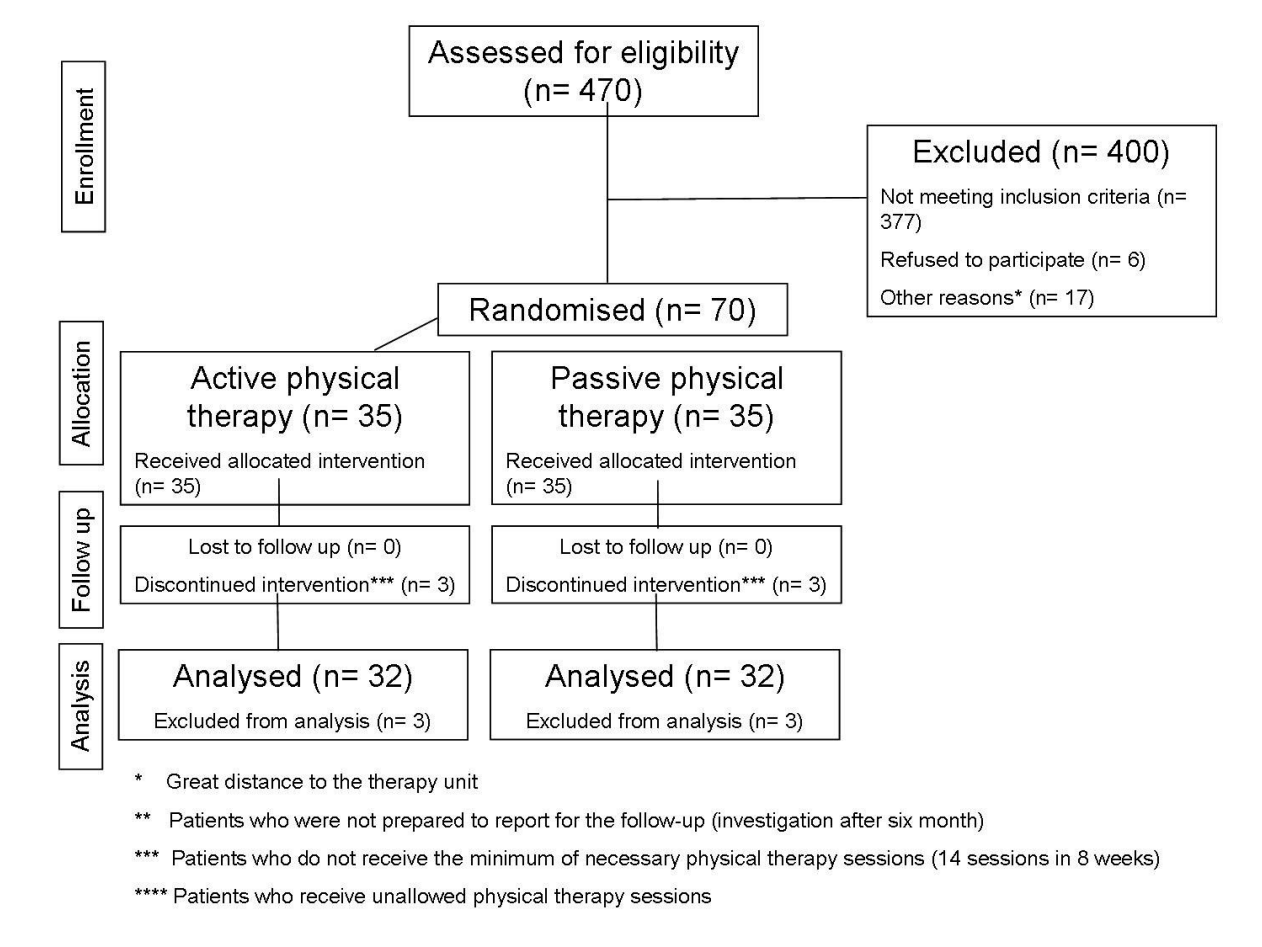
**Flow chart of the active and passive physical therapy group**.

Seventy patients were randomized to one of two groups treated with either "active physical therapy" (APT) or "passive physical therapy" (PPT) (figure 1). For the randomization process the method of a single block randomization was used. For each of the therapy modalities APT and PPT 35 envelopes were prepared, closed, mixed and finally numbered. After study inclusion of a patient the numbered envelope was opened corresponding to the order of enrolment and the patient assigned to one of the two therapy modalities. The results of these two groups were compared with those obtained in an earlier study of a collective of 20 patients to whom the recommendation "act as usual" (AUU) had been given. This latter group fulfilled the same inclusion criteria and underwent analogous diagnostic procedures and initial treatments as the two randomized groups.

### Study procedure

The study complied with applicable German law and with the principles of the Helsinki Declaration and was approved by the institutional ethics commission. All patients gave their written informed consent for study participation. Intake examination was performed within 24 hours after the whiplash trauma and assessed patients' pain score (PS) and range of motion deficit (ROMD) in the cervical spine.

For study inclusion, patients had to fulfill the criteria for QTF II whiplash injury (patients with neck pain and musculoskeletal signs) (table 1). Patients who had suffered previous injuries of the cervical spine or who had muscular, neurological or mental disorders were excluded from participation in the studies. Osseous injuries were excluded by appropriate radiographic imaging. Patients who had no deficit in range of motion at the intake examination were also excluded as classified as QTF I patients.

All patients were initially given a standard prescription for a non-steroidal anti-inflammatory agent (NSAID) to be taken for seven days twice a day and were provided with a soft cervical collar for the same duration. During this period, patients were asked to keep a record of how long the cervical collar was worn each day and how much of the analgesic medication they had used. After seven days patients returned the soft cervical collars, at which time their reported duration of collar use and NSAID intake were checked.

At this point, patients in the APT and PPT groups started a standardized physical therapy program provided through the clinic's physical therapy department three times per week for a period of seven weeks. Patients in the AAU group were informed in a detailed consultation session. Outcome parameters in all groups were reassessed after 8 weeks and additionally the period of disability (POD) was asked.

### "Passive" therapy

The treatment consisted of the application of moist heat [22,23], classic massage [22] and electrotherapy [22,24].

### "Active" therapy

#### Week 2

Soft-tissue treatment [25], trigger point treatment [22-24], joint mobilisation without involvement of the cervical spine, posture training and electrotherapy.

#### Week 3

As above, with addition of coordination training [26], training of the trunk and extremities and stabilization techniques with short, segmental leverage [23].

#### Week 6

As above, with addition of three-dimensional training with the head's own weight as the limit of resistance.

#### Week 8

As above, with addition of specific joint mobilization of the cervical spine, if necessary

### "Act as usual"

In a detailed consultation session, the benign nature of the injury was explained to the patients. Patients were given the recommendation to resume their usual activities without modification. No therapeutic measures were recommended.

### Pain Score

Patients' pain scores were determined using two visual analog scales, each 100 mm in length. Patients were asked to indicate their average degree of pain and their most severe pain, respectively. The pain score was calculated as the average of these two values. The change in pain from one assessment to the next is given as ΔPS.

### Range of Motion Deficit

To improve the information content of the individual deficit in range of motion, the mobility of the cervical spine was assessed for all six directions of motion (flexion, extension, rotation and lateral flexion) and these respective figures were combined to obtain a single sum. Motion was measured by the same investigator with a two-legged goniometer. For the flexion and extension measurements, the instrument was applied perpendicular to the connecting line between the angle of the eye and the tragus. For the determination of rotation, the tangential alignment was performed on the side of the cranium. The values were rounded to the nearest 5° for accuracy of measurement. This sum was then subtracted from pre-defined normal values (sum = 330°) and the difference was considered to represent a patient's deficit in range of motion. The change in the range of motion deficit from one assessment to the next is given as ΔROM.

### Period of Disability/Sickness costs

The POD in days was recorded by telephone after three to six months. Total sickness costs were calculated based on the costs of physical therapy and the patient's lost income due to missed time at work. Costs for therapy were based on the fees recognized in the official German fee schedule for the allied health professions. Costs for missed work were based on the average prevailing daily wage in Germany [27].

### Evaluation of data

Data was evaluated descriptively. Improvement in any given parameter was calculated as the difference in values before and after treatment. Results were tested for statistical significance using the Wilcoxon test for linked samples. The Wilcoxon test for linked samples was also used for the subgroup analysis of the AAU group. Because of the non-normal distribution of the values the data presentation was done using Min/Max and Median. Differences were considered statistically significance for values of p < 0.05.

## Results

### Subject participation

Of the 470 patients with distortion injuries of the cervical spine treated in the emergency department, 93 fulfilled the inclusion criteria. Six patients declined to participate and another 17 patients were unable to participate in the study due the distance from home to the therapy unit. The 70 remaining patients were randomized to the two physical therapy groups (n = 35; figure 1). Three patients in each group were excluded from the study because they failed to report for the follow-up examination after two months. A total of 32 patients per group could thus be evaluated in the APT (10 males, 22 females; median age: 28 years, range: 19–52 years) and the PPT (12 males, 20 females; median age: 29 years, range: 18–51 years) groups.

The AAU group consisted of a collective of 20 patients who had participated in a previous study. Six of the 20 recruited patients refused to report for the follow-up after two months. Seven patients who were evaluated after two months had undergone physical therapy in violation of the study protocol. Only seven patients in this group completed their participation correctly at two months. Of the 14 patients evaluated, five were males and nine were females (median age: 29 years, range 19– 50 years).

There were no differences between the three groups in terms of patients' age, gender distribution, consumption of NSAIDs or period of immobilization with a soft cervical collar (table 2).

**Table 2:** Patients data of the three study groups

	**APT**	**PPT**	**AAU**
**Participants**	32	32	14
Age*	28 (19–52)	29 (18–51)	29 (19–50)
**Gender**	10 male; 22 female	12 male; 20 female	5 male; 9 female
Consumption of NSAIDs**	7,0 (5,5–8,5)	7,0 (5,0–8,5)	7,0 (6,0–8,0)
Period of Immobilization**	7,0 (5,5–8,5)	7,0 (5,0–8,5)	7,0 (6,0–8,0)

APT: active physical therapy; PPT: passive physical therapy; AAU: act-as-usual; NSAID: non-steroidal anti-inflammatory drug; data are presented as median (min-max). * in years; ** in days

### Pain Score

In the APT group, there was a significantly greater median improvement in pain (ΔPS = 50.5) than in the PPT group (ΔPS = 39.2; p = 0.035) and the AAU group (ΔPS = 28.8; p = 0.009; table 3).

**Table 3:** Group analysis of the APT, PPT and AAU groups

	**APT**		**PPT**		**AAU**	
Parameter	Median	**Min/Max**	Median	**Min/Max**	Median	**Min/Max**
**PS_initial_**	57.2	**(34.5–88.0)**	56.7	**(28.0–81.5)**	57.5	**(7.5–85.0)**
**PS_post_**	4.7	**(0.0–52.2)**	15.7	**(0.0–66.5)**	20.0	**(0.0–67.5)**
**ΔPS**	50.5	**(0.0–88.0)**	39.2	**(5.5–69.5)**	28.8	**(-2.5–65.0)**
**ROMD_initial_**	120°	**(35–290°)**	108°	**(25–210°)**	100°	**(20–160°)**
**ROMD_post_**	0°	**(0–60°)**	0°	**(0–35°)**	10°	**(0–50°)**
**ΔROMD**	120°	**(35–290°)**	108°	**(5–210°)**	70°	**(20–160°)**
**POD**	14	**(0–56)**	14	**(0–56)**	49	**(14–80)**

APT: active physical therapy; PPT: passive physical therapy; AAU: act-as-usual; PS = pain score on VAS; ROMD = range of motion deficit in degrees (°); POD = period of disability in days

In the AAU group, the seven patients who underwent physical therapy achieved a median ΔPS of 37.5, while the seven patients who complied with the therapy recommendation to "act as usual" showed no median change in PS (table 4). There was no significant group difference (p = 0.513).

**Table 4:** Subgroup analysis of the AAU group (n = 14) comparing the non-compliant (n = 7) and compliant subgroups (n = 7).

	**Non-Compliant**		**Compliant**	
Parameter	Median	**Min/Max**	Median	**Min/Max**
**PS_initial_**	67.5	**(35.0–85.0)**	45.0	**(7.5–62.5)**
**PS_post_**	20.0	**(0.0–67.5)**	12.5	**(5.0–60.0)**
**ΔPS**	37.5	**(0.0–65.0)**	0.0	**(-2.5–55.0)**
**ROMD_initial_**	90°	**(20–160°)**	110°	**(40–140°)**
**ROMD_post_**	0°	**(0–30°)**	40°	**(0–50°)**
**ΔROMD**	80°	**(20–160°)**	70°	**(40–140°)**
**POD**	63	28–84	28	14–77

AAU: act-as-usual; PS = pain score on VAS; ROMD = range of motion deficit in degrees (°); POD = period of disability in days

### Range of Motion Deficit

At two months, all patients exhibited improvement in their respective ranges of motion (table 3). Both physical therapy groups as a whole showed no deficit in median range of motion while the median range of motion deficit in the AAU group was 10°. The median ΔROMD was 120° in the APT group and 108° in the group (p = 0.6473). In the AAU group, median ΔROMD was 70° with no a significant difference in comparison to either the APT (p = 0.062) or the PPT group (p = 0.071).

In the AAU group, the seven patients who underwent physical therapy achieved a median ΔROMD of 80° while the seven patients who complied with the therapy recommendation to "act as usual" showed a median ΔROMD of 70° (table 4). There was no significant group difference (p = 0.221).

### Period of Disability

In both physical therapy groups the median POD was 14 days compared to 49 days in the AAU group. There was a significant group difference compared to each of the two physical therapy groups (p = 0.001; table 3). Average total costs in the APT and PPT groups were € 4,129 and € 3,754, respectively compared with € 11,407 in the AAU group (table 5).

**Table 5:** Analysis of total costs for treatment and missed work (German fee schedule and average daily wages) for the APT, PPT and AAU groups

	**APT**	**PPT**	**AAU**
**Therapy costs**	21 × 41.40 € = **869.80 €**	21 × 23.60 € = **495.60 €**	--
**Missed work**	14 × 232.80 € = **3259.20 €**	14 × 232.80 € = **3259.20 €**	49 × 232.80 € = **11407.20 €**
**Total costs**	**4,129.00 €**	**3754.80 €**	**11407.20 €**

APT: active physical therapy; PPT: passive physical therapy; AAU: act-as-usual

In the AAU group, the seven patients who underwent physical therapy had a median POD of 28 days while the seven patients who complied with the therapy recommendation to "act as usual" had a median POD of 63 days; they differed significantly from the subgroup that underwent physical therapy (p = 0.003; table 4).

## Discussion

This study shows that the effectiveness of physical therapy and the recommendation to "act as usual" varies strongly in dependence of the outcome parameter we look at.

There was no statistically significant difference in the improvement of range of motion deficit of the cervical spine observed in the groups receiving active or passive physical therapy and the group receiving the recommendation to "act as usual". The effects of physical therapy on range of motion are discussed controversial in the literature. While a few studies (observation periods of 4–12 weeks) reported a significant improvement in range of motion in patients receiving physical therapy compared to a spontaneous clinical course [28-30], this was not confirmed by other studies (observation periods of 6–26 weeks) [21,31,32]. Because physical therapy appears to have only a limited effect in comparison with the spontaneous clinical course and limitations in range of motion resolve without treatment in many patients within eight weeks of the injury [29], it is understandable that differences between the various therapeutic options are difficult to demonstrate.

With respect to pain intensity, previous studies have shown that activity and physical therapy result in enhanced pain reduction compared with inactivity [19,21,28-30,33,34]. Analogous to our results, Mealy et al. showed that "active" physical therapy resulted in superior reduction in pain intensity than did "passive" physical therapy [29]. Furthermore, our results confirm the findings of two other studies that found a significantly better reduction in pain as a result of active physical therapy compared to the recommendation to "act as usual" [33,35]. Only Borchgrevink et al. consider the recommendation to "act as usual" to be sufficient [31]. In our study, the initial pain intensity of the AAU group was very low compared with the other groups. The subgroup analysis of the AAU group in our study suggests that patients with initially low pain intensity are willing to accept the recommendation "act as usual". Seen from this perspective, the data of the Borchgrevink study and those of the present study could be interpreted to mean that "act as usual" may be an adequate approach for QTF grade II populations with initially low pain intensity. On the other hand, whiplash patients with high pain intensity are not satisfied with the recommendation to "act as usual" and in many cases seek an alternate therapy prescribed by another physician or other healthcare provider. In the end, the patient acts on his own to "correct" the prescribed therapy but at the same time incurs additional healthcare costs.

Most of the financial cost of whiplash injuries relates to the resulting inability to work. Few studies, however, have addressed whiplash patients' period of disability [16]. In one study, Pennie et al. found no difference in the length of the period of disability between patients undergoing "active" physical therapy and those treated with simple immobilization of the cervical spine [20]. In agreement with the findings of the present investigation, however, two studies found that physical therapy significantly reduced the period of disability [19,21]. Since the two physical therapy programs in our study did not differ significantly in terms of their effect on the period of disability, it would appear that which type of physical therapy a patient receives is less important than the fact that some form of physical therapy is offered.

One limitation of this study is lack of randomization in the AAU group. The findings of the AAU group were obtained as part of a second, separate study using identical methods. The AAU collective did not differ in terms of socioeconomic level, age or gender distribution, accident history or severity of injury. Thus, use of the AAU collective for comparison would appear to be legitimate, although the results of the comparison between the AAU group and the two physical therapy groups would only meet criteria for EBM level 3.

## Conclusion

The findings of the present study show that the importance of physical therapy in patients with whiplash injuries varies significantly in relation to outcome parameter. Thus, a referral for physical therapy may not be considered medically necessary for restoring range of motion, yet it would appear to be extremely important from an economic standpoint in reducing patients' period of disability. From the patients' point of view, however, reduction in pain intensity is the most important goal. Here, prescription of "active" physical therapy should be preferred. Considering all these factors, active physical therapy is recommended for patients with QTF grade II whiplash injuries as the best option for achieving both therapeutic and economic objectives.

## Competing interests

The authors declare that they have no competing interests.

## Authors' contributions

CD drafted the manuscript. ME participated in the study coordination and helped in the medical examination. PS participated in the medical examination. MS performed the statistical analysis. FS carried out the physical therapy programs. GK helped to draft the manuscript. MK participated in the study design and its coordination. All authors read and approved the final manuscript.
